# Artificial intelligence for interpretation of segments of whole body MRI in CNO: pilot study comparing radiologists versus machine learning algorithm

**DOI:** 10.1186/s12969-020-00442-9

**Published:** 2020-06-09

**Authors:** Chandrika S. Bhat, Mark Chopra, Savvas Andronikou, Suvadip Paul, Zach Wener-Fligner, Anna Merkoulovitch, Izidora Holjar-Erlic, Flavia Menegotto, Ewan Simpson, David Grier, Athimalaipet V. Ramanan

**Affiliations:** 1 Paediatric Rheumatology Service, Rainbow Children’s Hospital, Bengaluru, India; 2 Department of Paediatric Radiology, Bristol Royal Hospital for Children, Bristol, BS2 8BJ UK; 3 Department of Paediatric Radiology, The Children’s Hospital of Philadelphia and University of Pennsylvania, Civic Centre Boulevard, Philadelphia, USA; 4 Stanford University, Stanford, California, USA; 5 Stanford University SCPD, Stanford, California, USA; 6 Translational Health Sciences, University of Bristol, Bristol, UK

**Keywords:** Whole body MRI, Pre- and post-pamidronate scan, Artificial intelligence

## Abstract

**Background:**

To initiate the development of a machine learning algorithm capable of comparing segments of pre and post pamidronate whole body MRI scans to assess treatment response and to compare the results of this algorithm with the analysis of a panel of paediatric radiologists.

**Methods:**

Whole body MRI of patients under the age of 16 diagnosed with CNO and treated with pamidronate at a tertiary referral paediatric hospital in United Kingdom between 2005 and 2017 were reviewed. Pre and post pamidronate images of the commonest sites of involvement (distal femur and proximal tibia) were manually selected (*n* = 45). A machine learning algorithm was developed and tested to assess treatment effectiveness by comparing pre and post pamidronate scans. The results of this algorithm were compared with the results of a panel of radiologists (ground truth).

**Results:**

When tested initially the machine algorithm predicted 4/7 (57.1%) examples correctly in the multi class model, and 5/7 (71.4%) correctly in the binary group. However when compared to the ground truth, the machine model was able to classify only 33.3% of the samples correctly but had a sensitivity of 100% in detecting improvement or worsening of disease.

**Conclusion:**

The machine learning could detect new lesions or resolution of a lesion with good sensitivity but failed to classify stable disease accurately. However, further validation on larger datasets are required to improve the specificity and accuracy of the machine model.

## Key messages

- When tested initially the machine learning model was able to classify half the test images accurately.
- On comparison with the ground truth, the machine model was able to classify only 33.3% of the samples correctly but had a sensitivity of 100% in detecting improvement or worsening of disease.

## Background

Chronic non-bacterial osteitis (CNO) is an auto inflammatory bone disorder characterised by the presence of sterile bone lesions. With increasing knowledge of the disease it has emerged that a whole body magnetic resonance imaging (WB-MRI) is a useful tool for diagnosis and also for assessing response to treatment [1, 2]. However, access to WB-MRI can be variable across different centres. Typical MRI (preferably Short Tau Inversion Recovery sequences (STIR)) non-specific features include bone marrow oedema, bone expansion, lytic areas and/or periosteal reaction [3, 4]. Analysing whole body MRIs can be time consuming as it requires extensive coverage of the whole skeleton and is also subject to diagnostic variability. Diagnostic confusion can occur in children with normal variants such as residual haematopoietic bone marrow or physiological stress response as well as organic pathologies such as lymphoproliferative disorders or infective osteomyelitis [5]. Developing a machine algorithm trained to compare scans pre and post treatment can be helpful in generating more consistent results.

Artificial intelligence is becoming increasingly popular in the field of radiology across various subspecialties. Deep learning with convolutional neural networks (CNNs) is gaining attention for its high performance in recognizing images. Recent studies have shown a performance level almost comparable to practicing radiologists [6–8]. For instance, deep learning had higher sensitivities but lower specificities than radiologists in classifying lymph node metastasis on PET-CT [9].

However, implementation of AI on a larger scale is largely limited by the amount of data available to train the algorithm. This is more apparent with rare diseases where automated labelling algorithms are virtually absent and only a limited number of human readers have expertise in such areas. None of the studies published so far have trialled deep learning in CNO. CNNs require large datasets which are difficult to obtain in rare diseases like CNO. For such diseases, alternatives like careful data augmentation, cross-validation and regularization can be used to reduce over fitting during machine training.

The objective of our study was twofold. The first aim was to develop a machine learning algorithm capable of comparing segments of pre and post pamidronate images derived from whole body MRI to assess treatment response and then validate the predictive model retrospectively on a randomly selected dataset of scans. The second goal was to compare the results of this algorithm with the ground truth. A panel of radiologists was formed to analyse the same set of images and the panel overall majority decision was considered the ground truth.

## Methods

Whole body MRI of patients under the age of 16 diagnosed with CNO at a tertiary referral paediatric hospital in United Kingdom between 2005 and 2017 were retrospectively reviewed. MRIs were acquired from clinical 1.5 T scanners and included coronal two-dimensional STIR T2 sequence images. Only those who received pamidronate were included in the study. WB MRI was usually performed at diagnosis and after completion of treatment to assess response. As a proof of concept study the pilot only utilised coronal images of the knee component of the WB MRI study, which is the commonest site of involvement in this disease. Also, these sites are relatively less complex to analyse in contrast to other areas such as wrists and ankles. Treatment response was ascertained by comparing each site against itself on the follow up scan. The MRI dataset was provided in DICOM format, each scan consisting of approximately one thousand individual images depicting cross-sections of the body. From this initial data, a pared-down dataset was manually curated by selecting one to two representative images with clear views of the knee and long bones of the leg from each MRI scan. In some cases, patients had no high-quality representative images because all leg and knee images were extremely blurry or noisy; these scans were omitted from the set. The retrieved dataset was augmented by swapping the order of pairs (pre and post pamidronate scans), and by using techniques such as Gaussian noise and random linear stretching to create more training images. Swapping order of pairs was done to leverage more data in order to help the system improve on performance and also to stabilize the behaviour of the model. Addition of Gaussian noise makes images more blurry and linear stretching magnifies an image by zooming in. These manoeuvres help the machine learning model become more robust to detect changes by telling it automatically which areas to focus on without really impacting the images.

Disease sites of the training and test samples were labelled using OsiriX DICOM viewer software and this was performed by a single radiologist for standardisation purposes. Disease progression labels were manually curated based on the information provided by this index radiologist. A machine learning algorithm was developed to assess treatment effectiveness by comparing pre and post pamidronate scans and classify them as:

- ‘Improved’ - when there was a reduction in number of lesions or decrease in signal intensity of existing lesions and there were no new lesions.
- ‘Regressed’ or ‘Worse’- when there were new lesions or increase in signal intensity of existing lesions.
- ‘Stable’ or ‘Persistent’ - when there was no change in signal intensity and there were no new lesions.

**Fig. 1:**
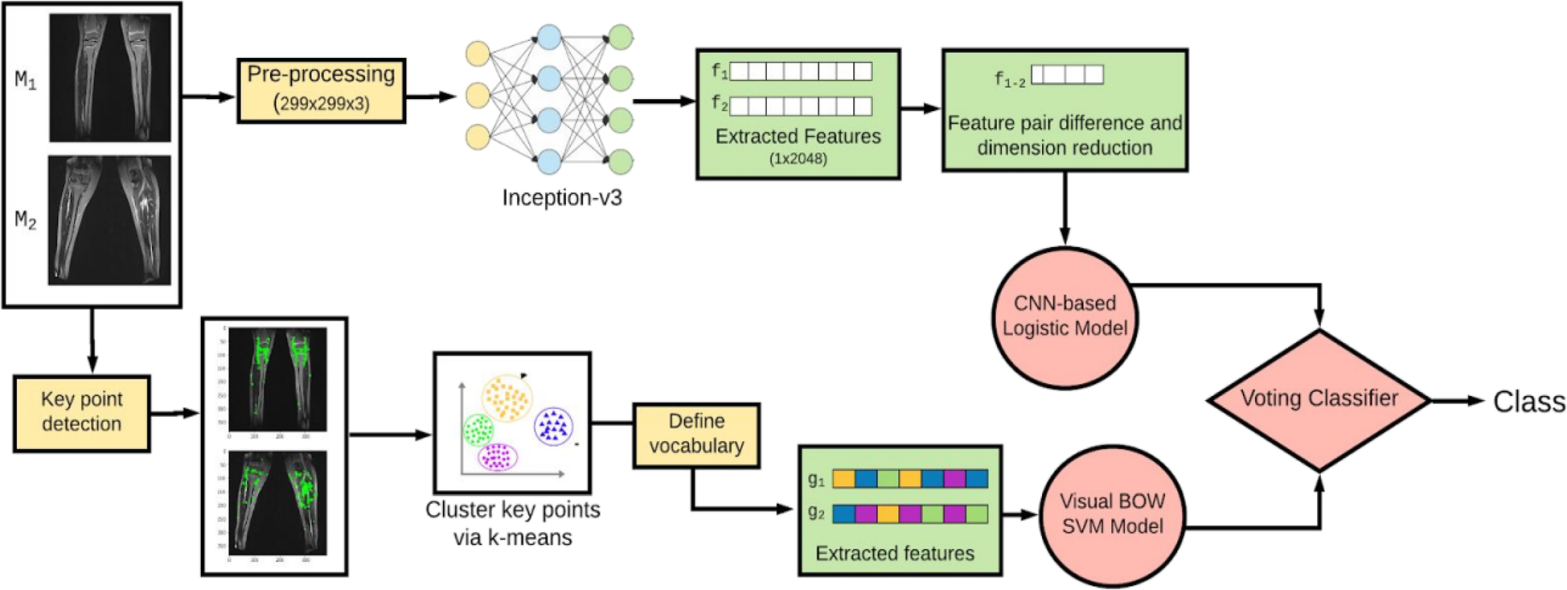
Overall architecture of the proposed machine algorithm model

The machine learning model comprises of two components followed by an ensemble method. An ensemble method combines the predictions of multiple models (in this case two models) either simply or by smartly weighting each component fed to it. In this paper we have used soft weighting, which considers the probabilities produced by component models, whereas hard voting considers only the predicted class. The algorithm has been summarised in Fig. 1. Each component is a machine learning model. The upper component extracts features, embeddings and representations from processed images using a pertained network. The specific pre-trained network used is mentioned in Fig. 1. Pre-training refers to using large models which have been trained on general data (and these are extremely popular models), for example if we wanted to learn the representation of a cat vs. a dog, it would help if we were able to convert these actual images into abstract representations of each other such that in the new space all images of cats are close to each other and far away from all images of dogs (and vice versa). After learning these representations from the image we feed this into a linear logistic model to produce a probability score. This output is fed into the ensemble. The lower component uses clustering to produce embeddings. These embeddings are the clusters which each image belongs to. Clustering is an unsupervised method which finds similar images in a dataset and labels them to be belonging to the same cluster. These clustering methods are only performed on processed images by popular image processing techniques such as Speeded Up Robust Features (SURF) and Scale Invariant Feature Transform (SIFT). This process is called a bag of visual words. This representation is fed into a Support Vector Machine (SVM) to produce a probability score. SVM is a supervised machine learning algorithm which can be used for both classification or regression challenges. When provided with training data a line which divides a plane into different parts is generated and on either side of this line lie different classification groups. This output is also fed into the ensemble.

In the first half of the exercise, the algorithm was tested on a dataset of seven pairs of images which were different from those used for training the machine model. Results were compared to the disease progression labels derived from the assessment of the index radiologist. Multi-class and binary models were generated for each of the independent methods used to develop the algorithm. The multiclass model classified scans as improved (I), regressed/worse (R) or stable (S) separately whereas the binary model grouped regressed and stable together.

**Fig. 2:**
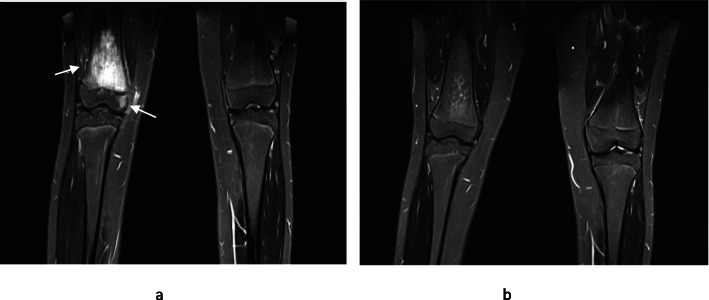
Pre and post pamidronate treatment MR images. Pre and post- pamidronate WB-MRI images of a 15 year old girl who presented with significant right knee pain and was diagnosed with CNO following a bone biopsy. Her symptoms resolved completely following four cycles of pamidronate. 2a – The coronal STIR MR image shows extensive high signal predominantly of the distal right femoral metaphysis consistent with intra-osseus oedema. A smaller area of the medial epiphysis is affected without features of cortical destruction or significant soft tissue component. 2b – Almost complete resolution of the metaphyseal high signal is in keeping with treatment response. The epiphyseal component is also no longer visible. In our exercise, the machine algorithm and panel of radiologists concurred that lesions resolved post treatment

In the second half of the exercise, five radiologists (panel of four radiologists from our hospital and one independent radiologist) were asked to review the same test images using a checklist and classify pre and post treatment scans as improved, regressed or stable (Fig. 2). The index radiologist was excluded from this exercise. All the radiologists were blinded to clinical information. The frequency of panel findings was then calculated. Inter-observer agreement was calculated using the Fleiss kappa coefficient. A kappa score of more than 0.6 was taken as consensus and this was considered as ground truth. Results of the machine learning algorithm were then compared to the ground truth. Statistical analysis was performed using Microsoft Excel version 12.0.

## Results

**Fig. 3:**
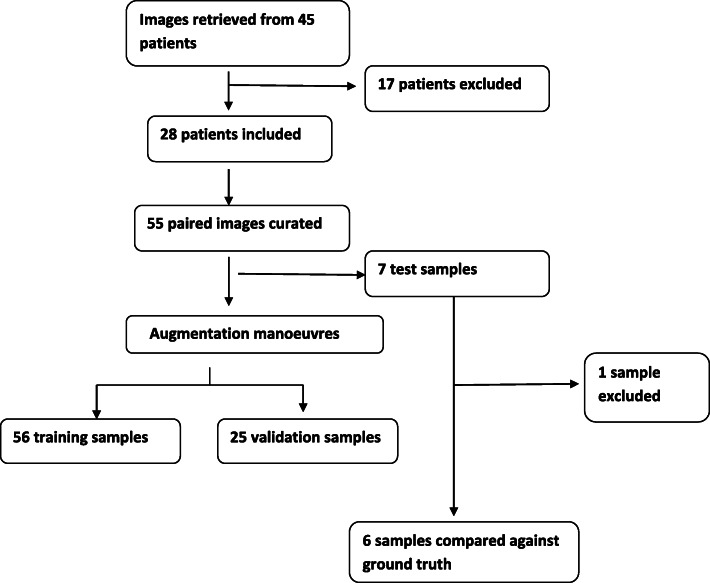
Summary of data collection

Images of the knee (including the distal end of the femur and proximal tibia) derived from WB MRI of 45 patients treated with pamidronate were retrieved. Scans of poor quality were excluded from the initial dataset leaving scans of 28 patients. From this dataset, 55 pairs of images were manually curated of which 7 test samples were hand -selected for assessing final model quality at the end of development. The test samples did not overlap with those in the development set. The remaining 48 samples were amplified. This augmented data set included 56 training samples and 25 validation samples. In the second half of the exercise, one sample was excluded from the test sample (since 2 of the 7 test samples were of the same patient), reducing the number to 6. Data collection has been summarised in Fig. 3.

- *Results of the machine learning model:* The ensembled models predicted 4/7 (57.1%) examples correctly in the multi class model, and 5/7 (71.4%) correctly in the binary group. Consistently, all multi-class models were unable to properly predict class S (stable). Area under curve (AUC) for Class I was 0.89, Class R was 0.91 and Class S was 0.68. Scan interpretations by radiologists and the machine learning algorithm have been summarised in Table 1.
- *Results of comparison of machine learning model* vs. *ground truth:* Results have been summarised in Table 2. The machine learning model was able to classify 2/6 (33.3%)examples correctly.

**Table 1:** Classification of scans by radiologists and machine learning algorithm

Serial Number	InRa^a^	Machine Interpretation	Re^b^ 1	Re 2	Re 3	Re 4	Re 5	Kappa coefficient	Consensus
1	I	R	S	R	S	S	S	0.6	S
2	S	R	S	I	S	S	S	0.6	S
3	R	R	S	S	S	S	S	1	S
4	I	I	I	I	I	I	I	1	I
5	R	R	R	R	R	R	R	1	R
6	S	I	S	S	S	S	S	1	S

^a^Index Radiologist, ^b^ Reader

**Table 2:** Comparison of machine learning algorithm against the ground truth

		Machine Algorithm Value
Sensitivity (%)	Improved	100
	Regressed	100
	Stable	0
Specificity (%)		40
Positive Likelihood Ratio		1.67
Negative Likelihood Ratio		0
Positive Predictive Value (%)		25 (27.3 to 72.7)
Negative Predictive Value (%)		100
Accuracy (%)		33.3

## Discussion

The machine model was able to classify half the test images correctly in the first half of this exercise. However, results of the multiclass group were not at par with the binary group. This may be due to a smaller number of samples in the ‘stable’ class, or to difficulty extracting features representative of this class compared to ‘improved’ or ‘regressed’ classes which had a better AUC.

In the second half of the exercise, the machine model was able to classify only 33% of the examples correctly but demonstrated a high sensitivity (100%) in detecting improvement or worsening of disease. However specificity (40%) and accuracy (50%) were low. In particular, the model failed to classify stable disease correctly. This may be due to their poor ability in recognising normal variants that can produce signal changes similar in appearance to CNO as there is no ground truth relating to radiology that states a bright lesion on MRI is CNO. For example in other parts of the body such as hands and feet bright signals can be considered as normal [10, 11].

Our study has a few limitations. The dataset was quite small and was further split to create two groups for machine training and testing. Acknowledging the small sample size, our results are only an indication of the performance of the machine algorithm and larger studies in collaboration with other centres are required to validate these observations. Only images around the knees and not the whole body were included. Hand selected images may not be representative of the actual labels generated from the WB-MRI. This can be attributed to the difference between static images and a stack or sequence of images. Also, images used for machine training were annotated by only one radiologist. Further research is needed to expand the study to whole body MRIs, to validate the model prospectively in real time and to determine its utility in clinical setting.

## Conclusion

This is but a small step towards a developing a potentially useful technology that may assist radiologists in many different multifocal disease entities currently diagnosed with WBMRI. However, further research is needed to expand the study to whole body MRIs, to validate the model prospectively in real time and to determine its utility in clinical setting.

## Data Availability

The datasets used and/or analysed during the current study are reproducible and available at https://github.com/annamerk/crmo-diagnosis-using-mri/.

## Abbreviations

CNO: Chronic non-bacterial osteitis
WB MRI: Whole Body Magnetic Resonance Imaging
STIR: Short Tau Inversion Recovery
CNN: Convolutional Neural Networks
SURF: Speeded Up Robust Features
SIFT: Scale Invariant Feature Transform
SVM: Support Vector Machine
AUC: Area Under Curve
